# Psychometric validation of the household food insecurity access scale among Inuit pregnant women from Northern Quebec

**DOI:** 10.1371/journal.pone.0178708

**Published:** 2017-06-14

**Authors:** Lisa Teh, Catherine Pirkle, Chris Furgal, Myriam Fillion, Michel Lucas

**Affiliations:** 1 Psychology Department, University of Hawai`i at Mānoa, Honolulu, Hawaii, United States of America; 2 Office of Public Health Studies, University of Hawai`i at Mānoa, Honolulu, Hawaii, United States of America; 3 Indigenous Environmental Studies and Sciences, Trent University, Peterborough, Ontario, Canada; 4 Population Health and Optimal Health Practices Research Unit, CHU de Québec – Université Laval, St-Sacrement Hospital, Québec, Québec, Canada; 5 Department of Social and Preventive Medicine, Université Laval, Québec, Québec, Canada; TNO, NETHERLANDS

## Abstract

**Background:**

Globally, food insecurity is a major public health concern. In North America, it is particularly prevalent in certain sub-groups, including Indigenous communities. Although many Indigenous and remote communities harvest and share food, most food security assessment tools focus on economic access. This study describes the psychometric evaluation of a modified Household Food Insecurity Access Scale (HFIAS), developed for mixed economies, to assess food insecurity among pregnant Inuit women.

**Methods:**

The HFIAS was administered to 130 pregnant women in Nunavik (Arctic region of Quebec), Canada. Data were fit to a Rasch Rating Scale Model (RSM) to determine the discrimination ability of the HFIAS. Person parameter (Theta) estimates were calculated based on the RSM to provide a more accurate scoring system of the modified HFIAS for this population. Theta values were compared to known correlates of food insecurity.

**Results:**

Comparative fit indices showed preference for a modified version of the HFIAS over the original. Theta values displayed a continuum of severity estimates and those values indicating greater food insecurity were consistently linked to known correlates of food insecurity. Participants living in households with more than 1 hunter (Theta = -.45) or more than 1 fisher (Theta = -.43) experienced less food insecurity than those with no hunters (Theta = .48) or fishers (Theta = .49) in their household. The RSM indicated the scale showed good discriminatory ability. Subsequent analyses indicated that most scale items pertain to the classification of a household as moderately food insecure.

**Conclusions:**

The modified HFIAS shows potential for measuring food insecurity among pregnant women in Nunavik. This is an efficient instrument that can inform interventions targeting health conditions impacting groups that obtain food through both monetary and non-monetary means.

## Introduction

Numerous recent studies, both representative and population-specific, document alarmingly high rates of food insecurity among Indigenous Canadians, especially among the Inuit.[1] Canadian Inuit living in Nunavut have the highest recorded prevalence of food insecurity among Indigenous populations in developed countries.[2] Concerns have been raised that currently accepted definitions of food insecurity and the assessment instruments based on these definitions might not accurately fit the unique Canadian Inuit food system.[3] In Nunavik, estimates of food insecurity vary between 24%-74% depending on the instruments used and the sub-population examined.[4] Research has linked food insecurity in Canadian Inuit populations to various indicators of poverty including: household crowding,[5, 6] lower levels of education,[7] low income,[5] less educational achievement,[7] and no hunters in the family.[5, 7]

Reliable, valid, and culturally appropriate assessment tools are essential to adequately address food insecurity in this population.[3] Pregnant women are a particularly important sub-population for whom accurate food security assessment instruments are needed because of the implications of food insecurity on the health of future generations.[8] Such tools can inform interventions directed at increasing food security among pregnant women in Nunavik.[9, 10]

Most food security assessment tools involve structured face-to-face interviews.[10] Several aspects of reliability and validity need to be demonstrated in order to determine the extent to which a questionnaire accurately assesses the latent variable it purports to measure.[11] These include internal consistency, content validity, and construct validity. Internal consistency evaluates the degree to which items in the questionnaire measure the same construct.[11, 12] However, internal consistency can be inflated if items in the questionnaire are redundant.[11] Construct validity describes the extent to which a questionnaire assesses a construct and does not measure unrelated constructs.[11, 13] Content validity evaluates the degree to which a questionnaire addresses all components of the construct. If considerable differences in a construct, such as food insecurity, exist across cultures,[3] estimates of reliability and validity for a scale might not be retained in different populations.[14] In such cases, it is recommended that researchers field reevaluate its reliability and validity in the targeted population.[14]

The measurement of food insecurity among Indigenous Canadians, especially the Inuit, who rely partially on subsistence methods to procure food, has been subject to controversy.[3] In Inuit populations, such as that of Nunavik, there is a strong culture of sharing food among family and friends and harvesting food from the environment. [3, 15, 16] Such cultural practices weaken the association between monetary resources and food accessibility for inhabitants of Inuit communities. The most commonly used and validated food security assessment tool is the U.S. Household Food Security Survey Module (US HFSSM), developed by the United States Department of Agriculture (USDA) and adapted by Health Canada. The US HFSSM was initially developed to inform food security interventions in the United States by measuring food security accurately and efficiently at both nationwide and statewide levels.[17] A major limitation of the US HFSSM is that the items specifically enquire about monetary access to food (e.g. “did you/ or other adults in your household ever not eat for a whole day because there wasn’t enough money for food”). In a mixed food system, where food is available through alternative means, an exclusive focus on monetary access to food may over-estimate food insecurity by not accounting for other ways to obtain food. This is a gap in food insecurity research in Nunavik, and many other Indigenous regions of the Canadian North, where food can be procured through multiple streams of access including: purchasing through the formal market system, informal selling and trading, harvesting from the environment, and sharing.[15]

As a partial response to this limitation of the US HFSSM, especially in resource-constrained settings, the Food and Nutrition Technical Assistance III (FANTA) Project developed the household food insecurity access scale (HFIAS). According to the United Nations Standing Committee on Nutrition, this is the only tool to directly measure food insecurity as it does not explicitly mention monetary access[18] (e.g. “were you not able to eat the kinds of food you preferred because of a lack of resources?”). As such, it is theoretically better equipped to measure food security, and not only monetary access to food, in Nunavik. The HFIAS also contains a sub-scale known as the Household Hunger Scale (HHS), which purportedly measures the most extreme end of food insecurity: hunger. Both scales have demonstrated acceptable validity within a number of different cultural contexts but have yet to be validated in an Inuit population.[19–21]

To date, no published study has assessed the item-level psychometric properties of any food security scale used in a Canadian or United States Indigenous population. Several studies have provided evidence of convergent validity for the US HFSSM,[6, 22, 23] and one study examined the sensitivity and specificity of a single item on the US HFSSM.[24] Given a recent explosion of literature examining food security among Northern Indigenous populations,[2, 5, 6, 22, 25–27] especially the Inuit, there is an urgent need to validate food insecurity scales in Canada. This is reinforced by the acknowledged crisis of food insecurity in Northern Canada and the need to develop interventions and policy informed by accurate estimates of the prevalence and severity of food insecurity.[1] The urgency to accurately evaluate food insecurity in Northern Canada is accentuated among pregnant women, given the links between food insecurity during pregnancy and indicators of child development, such as birth weight and stature, in other populations. [28–30]

This study aims to increase the utility of the HFIAS in measuring food insecurity among pregnant women in Nunavik by understanding its content and construct validity in this population. A second objective of this study is to improve the accuracy with which we can identify pregnant Inuit women most in need of food security interventions using statistical modeling of HFIAS items. Finally, the current study attempts to construct a culturally-sensitive, empirically-based scoring system to assist with future research.

## Materials and methods

### Data collection

This study utilized baseline data from a longitudinal study examining the effectiveness of the Arctic Char Distribution Project (AC/DP); a culturally sensitive food security intervention for pregnant women in Nunavik. The AC/DP was conducted in collaboration with the Nunavik Regional Board of Health and Social Services between September 2013 and April 2014 and was approved by the Laval University ethics committee. Written consent was obtained from all participants. Details about the AC/DP are described elsewhere.[31] A total of 130 pregnant women living in Nunavik participated in the study. The mean gestational weeks of participants at enrollment was 16.4 (SD = 6.5) with 40 participants in their first trimester and 90 after their 13^th^ week.

Food insecurity was measured at an individual level among participants using the HFIAS, slightly adapted for the Nunavik population based on feedback from local partners (see S1 File for the questionnaire). The “Worry” (did you worry that you would not have enough food?) and “Lack Resources” (Were you not able to eat the kinds of food you preferred because of a lack of resources) items were designed to measure mild food insecurity. (23) Items measuring “Limited Variety” (did you have to eat a limited variety of foods due to a lack of resources) and “Unwanted Food” (did you have to eat some foods that you really did not want to eat because of a lack of resources to obtain other food items you prefer?) were purported to address mild to moderate food insecurity. “Reduced Meal Size” (did you have to eat a smaller meal than you wanted because there was not enough food available for you?) and “Reduced Meal Frequency” (did you have to eat fewer meals in a day because there was not enough food?) items were designed to measure moderate to severe food insecurity. Any level of endorsement of the “House Empty” (was your house ever out of food of any kind because of a lack of resources to get food), “Sleep Hungry” (did you go to sleep at night hungry because there was not enough food), or “Whole Day Without Eating” (did you ever go a whole day and night without anything to eat because there was not enough food in your house?) items originally classified participants as severely food insecure. (32)

The HFIAS is a 9-item questionnaire that assesses the severity of household-level food access deficiencies; it is designed to track the effectiveness of food security interventions internationally.[32] The HFIAS has demonstrated acceptable test-retest reliability and convergent validity based on socioeconomic status, and internal consistency (α = 0.80 to 0.91) in Lebanon[21] and Tanzania.[20] In one study, participating in an urban agriculture program was associated with mild improvement in food insecurity measured by the HFIAS.[19]

### Statistical analyses

Chronbach’s alpha describes the internal consistency of a scale.[12] This was computed as a preliminary assessment of the degree to which the HFIAS appears to measure the same latent construct. This was further supported by a principal component analysis conducted on the HFIAS data (see S1 Table and S1 Fig for a summary of those results).

Rasch rating scale model (RSM) is an item response theory (IRT) method designed to analyze data from measures using more than two ordinal response categories for each item, such as the HFIAS. Rasch models, including the RSM, allow for measurement of a person’s position along a continum of a latent variable, known as the person parameter, independently of the difficulty parameters, which describe the relative likelihood of various responses to each item.[33] This allows for a more accurate assessment of the construct validity of a rating scale than traditional scoring systems. Compared to other polytomous Rasch models, RSM is more appropriate for use in small samples.[34] Another strength of IRT models, including RSM, is that they allow one to determine the relative contribution of each item to information obtained from the scale along a continuum of the latent variable measured.[35] We conducted a one-parameter RSM analysis on the HFIAS data.

Difficulty parameters were estimated with STATA Data Analysis and Statistical Software[36] using Gaussian random effects method, which describes the level of food insecurity expected for each response while minimizing the difference between that and the observed levels.[37] Item information curves provide a visual representation of the difficulty parameters, based on the results of IRT analyses.[35] In health care research, they can demonstrate the level of severity each item addresses. If the scale demonstrates a high level of content validity, it should provide information about the entire spectrum of food security/insecurity.

Infit mean square values (MSV) for each item can be calculated with Rasch models to understand the degree to which the scale appears to be unidimensional.[38] We calculated the infit mean square values for the HFIAS using the Extended Rasch Modeling package for R.[39] Unidimensionality refers to the extent to which items on a scale measure a single latent construct, such as food security.[40] Infit MSV over 1.4 are considered to represent items that introduce too much statistical noise into the model and MSV below 0.6 indicate limited information is provided by the item due to high inter-item correlations. If the scale is unidimensional, infit MSV should fall within acceptable ranges. Two items were identified as potentially problematic due to cultural concerns. The RSM was run with these items removed sequentially. If the scale displays good discriminant validity, removing items should not improve its fit.

Akaike information criterion (AIC) and Bayesian information criterion [41] are indices of comparative model fit.[42] They indicate the relative amount of unmodeled variance provided by different statistical models. Lower AIC and BIC values indicate a preferred model. AIC and BIC indices were calculated for the RSM utilizing the original HFIAS scale and for the RSM of the HFIAS without the two potentially problematic items.

Person parameter (Theta) values, which are estimates of the latent trait experienced by each individual[43], in this case food insecurity, were calculated to provide a more accurate estimate of food security severity compared to the HFIAS original scoring procedure. Theta values are estimated independently of item parameters, which estimate the degree to which an individual’s responses on a scale are attributable to item characteristics, such as difficulty, rather than the latent variable.[44] Theta values cannot be calculated for the lowest and highest raw scores. These scores were imputed using Baysian estimation (see Baker & Kim 2004 Chapter 7 for description of this method[45]). Theta values are reported for demographic groups predicted to show differences in levels of food insecurity.

## Results

Table 1 presents the demographic and socioeconomic characteristics of the sample and mean estimates of person parameters for different levels of each variable. Participants’ ages ranged from 18 to 43 with a mean age of 24.8 (SD = 5.3). More food insecure than the mean was noted among women at both ends of the sample’s age distribution, those with low formal educational attainment, who were in a domestic partnership, had three or more adults and/or children in the household, were unemployed, and who had few household hunters/fishers. Additionally, those from the Hudson Coast were more food insecure than those from the Ungava Coast. Women who drank alcohol, smoked cigarettes or cannabis during pregnancy were more food insecure than those who had not.

**Table 1:** Demographic and socioeconomic makeup of the sample.

	Frequency (%)	Mean Food Insecurity Score/Theta (SD)	More (+) or Less (-) Food Insecure than Mean responses^1^
**Overall**	**130**^2^	**-1.10 (1.91)**	
**Location**			
Hudson coast	71 (54.6%)	.28 (2.00)	+
Ungava coast	59 (45.4%)	-.24 (1.77)	-
**Age**			
≤25	84 (64.6%)	.14 (1.90)	+
26–30	27 (20.8%)	-.54 (1.61)	-
≥31	19 (14.6%)	.44 (2.25)	+
**Education**			
Partial secondary school or less	87 (66.9%)	.19 (1.92)	+
Completed secondary school or more	43 (33.1%)	-.24 (1.87)	-
**Civil Status (n = 128)**^2^			
Married/Domestic Partnership	101 (78.9%)	.10 (1.92)	+
Single	27 (21.1%)	-.21 (1.80)	-
**Living Arrangements**			
Adults (in the household)			
≤2	83 (63.9%)	-.04 (1.95)	-
≥3	47 (36.2%)	.18 (1.86)	+
Children (in the household)			
≤2	75 (57.7%)	-.26 (1.78)	-
≥3	55 (42.3%)	.46 (2.02)	+
**Employment Status (n = 130)**			
Currently working	52 (40.0%)	-.24 (1.80)	-
**Lifestyle indicators**			
Hunter (in the household)			
No	36 (37.1%)	.48 (1.89)	+
1	39 (40.2%)	.34 (1.98)	+
>1 hunter in the household	55 (42.3%)	-.45 (1.80)	-
Fisher (in the household)			
No	39 (30.0%)	.49 (1.78)	+
1	28 (21.5%)	.49 (2.15)	+
>1	63 (48.5%)	-.43 (1.79)	-
**During pregnancy**			
Current smokers	116 (89.2%)	.15(1.94)	+
Cannabis	43 (33.1%)	.35 (2.10)	+
Alcohol (n = 129)	57 (44.2%)	.05 (1.80)	+

^1^ Mean responses, excluding those with a raw score of 0 (the minimum) or 12 (the maximum), have a Theta of 0. Positive Thetas represent scores above the mean. Negative responses indicate scores below the mean. Responses with raw scores of 0 or 12 were imputed using Bayesian estimation. Because there were more participants with raw scores of 0 (N = 44, 33.8%) than 12 (N = 1, 0.7%), the overall mean scores are below 0. A constant of 1.1 was added to each score to create a mean of 0. This column reports whether scores were above or below the mean including the imputed scores. Thus, positive scores indicate more food insecurity while negatives scores indicate less food insecurity.

^2^ Missing values; the n is indicated when there are missing values.

**Fig 1:**
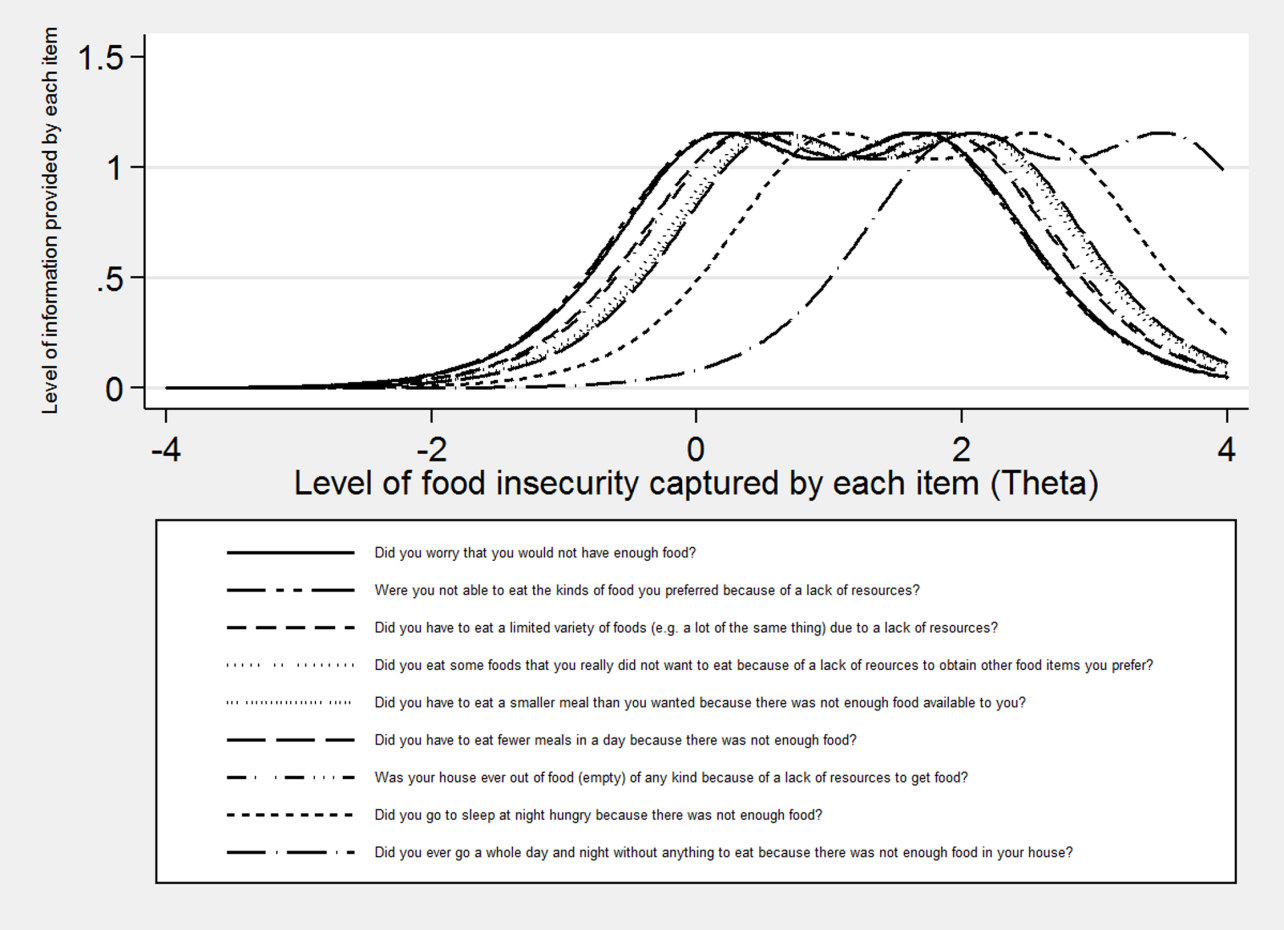
Item information function curves based on the RSM for the original HFIAS.

Fig 1 displays the information provided by each item along different levels of the latent variable; also known as the items’ difficulty parameters.[43] The item information function curves show that the HFIAS provides information along a continuum of the latent variable, presumed to be food insecurity. Each curve represents the level of food insecurity severity, located along the X-axis, measured by each respective HFIAS item. The first peak in each bimodal curve displays the probability that a person who sometimes experiences the event will respond “sometimes” to the item.[44] The second peak represents the probability that a person who often experiences the event will respond “often.” The Y-axis displays the average level of information provided by each item in the scale.[44] The House Empty item was initially proposed to tap into severe food insecurity,[32] but it appears to measure moderate food insecurity best in this sample.

**Fig 2:**
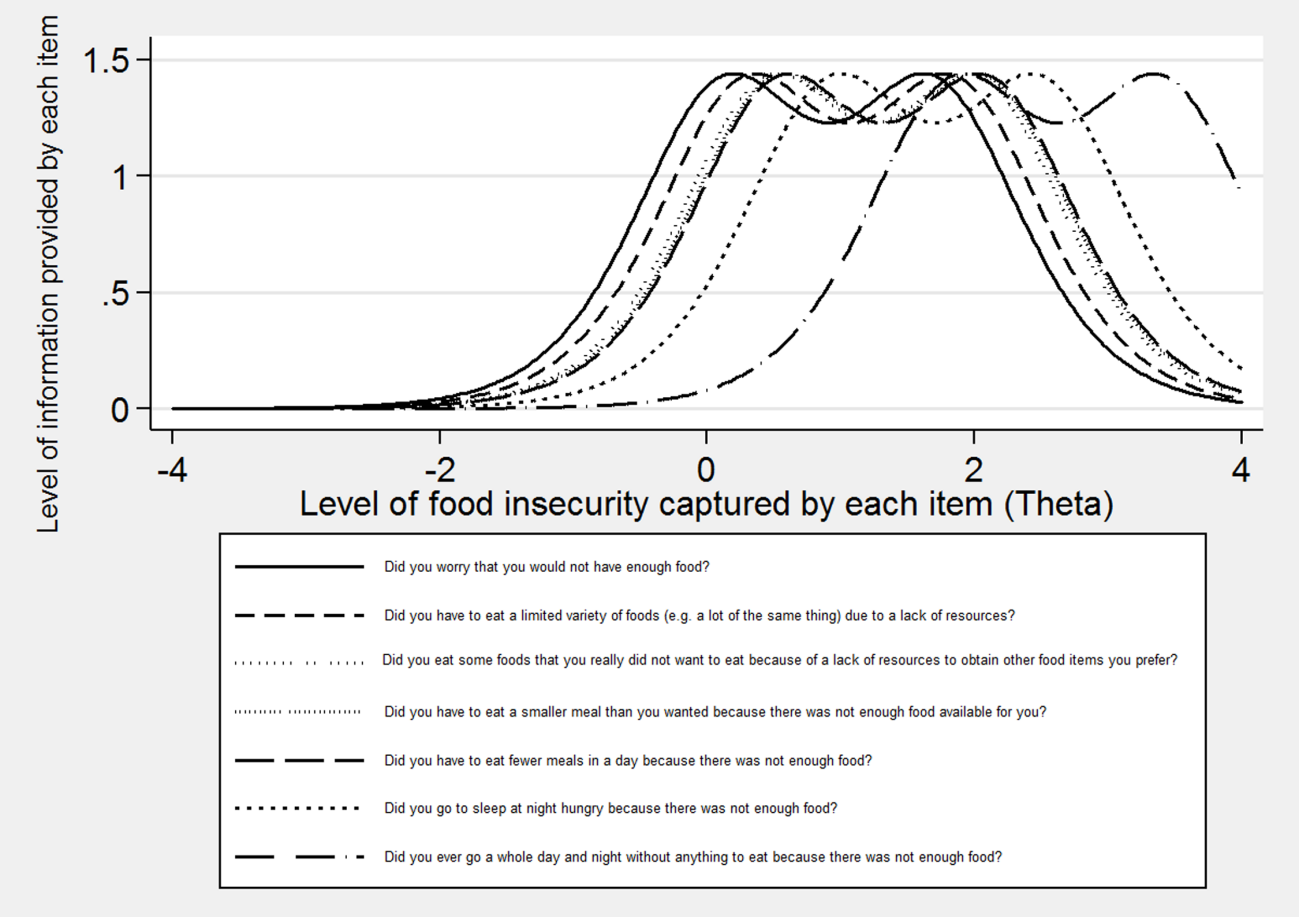
Item information function curves based on the RSM for the modified HFIAS.

Fig 2 is similar to Fig 1 and shows that the level of information provided by the HFIAS in this sample increased once the Lack Resources and House Empty items were removed. The Y-axis shows that the average level of information provided by the scale increased when the items were removed, indicating that the redundancy was reduced.

Table 2 shows that the items on both the original and a modified version of the HFIAS demonstrate acceptable infit MSV values. This indicates that both versions of the HFIAS are likely unidimensional. Chronbach Alpha’s for the original (α = .88) and modified (α = .86) versions of the HFIAS within the “acceptable” range (above .70).[12] The lower AIC and BIC values show a preference for the modified model over the original in terms of comparative information loss. The coefficient values from the Gaussian estimation, describing the difficulty parameters of each response, are consistently higher for 2 (often) versus 1 (sometimes) compared to 1 (sometimes) versus 0 (never).

**Table 2:** Infit values for each item in the original and modified version of the HFIAS and the coefficients for differences between response categories based on the RSM.

Item	Original scale infit MSV	Modified scale MSV		
		infit MSV	Coefficient (P)^1^	
			2 vs 1	1 vs 0
Did you worry that you would not have enough food?	.886	1.075	1.72 (<.01)	.09 (.52)
Were you not able to eat the kinds of food you preferred because of a lack of resources?	1.046	N/A	1.89 (<.01)	.26 (.07)
Did you have to eat a limited variety of foods (e.g. a lot of the same thing) due to a lack of resources?	.969	.948	2.06 (<.01)	.42 (<.01)
Did you eat some foods that you did not want to eat because of a lack of resources to obtain other food items you prefer?	.914	.884	2.11 (<.01)	.48 (<.01)
Did you have to eat a smaller meal than you wanted because there was not enough food available for you?	.911	.878	2.11 (<.01)	.48 (<.01)
Did you have to eat fewer meals in a day because there was not enough food?	.727	.667	2.14 (<.01)	.50 (<.01)
Was your house ever out of food (empty) of any kind because of a lack of resources to get food?	1.041	N/A	N/A	
Did you go to sleep at night hungry because there was not enough food?	.784	.763	2.53 (<.01)	.90 (<.01)
Did you ever go a whole day and night without anything to eat because there was not enough food in your house?	1.283	1.313	3.45 (<.01)	1.81 (<.01)
Chronbach’s α	.88	.86		
**Scale AIC**^2^	1523.718	1153.086		
**Scale BIC**	1555.261	1178.894		

^1^ 0: Never; 1: Sometimes; 2: Often; higher coefficients represent better discrimination between response categories

^2^ AIC: Akaike information criterion; BIC: Bayesian information criterion; MSV: Mean Square Value. Lower AIC and BIC values indicate a preferred model.

## Discussion

### Summary of main findings

The HFIAS demonstrated unidimensionality and promising construct validity in this sample. However, the modified model without two items outperformed the original model. The House Empty item was designed to measure severe food insecurity, yet appears to capture mild to moderate food insecurity in this sample. This may be due to cultural factors regarding food sharing between households in Nunavik.[15] Individuals who do not have food in their houses can often access food from a friend or family member’s house. As such, this item does not necessarily tap into a severe lack of access to food in this sample.

The demographic variables most strongly associated with food security were age, having more than two fishers in the household, and having more than two hunters in the household. This substantiates the findings from other studies among Canadian Inuit populations suggesting that hunting and fishing, as means of providing families with country food and/or with supplementary income, are strong determinants of food security.[5, 7] As such, this further demonstrates the need to collect data on access to country food in future measurements of food security among Inuit populations. One unique finding was women who were married or in a domestic partnership were more food insecure than single women. This could be explained by crowding. [6]

Across all items, the scale was consistently able to differentiate between individuals experiencing each symptom “often” versus “sometimes” better than between those experiencing the symptoms “sometimes” versus “never”. This is unusual and might point towards ambiguity in understanding the response categories. It is often recommended that scales have an even number of categories, as participants frequently choose the middle option to avoid committing to a response.[46] The word “never” in questionnaires can also bias responses, as people often are reluctant to endorse absolutes, particularly when discussing high base-rate situations, such as the mild to moderate items on the HFIAS.

### Comparisons with the literature

This is the first study to examine item-level psychometric properties of a food security questionnaire among an Indigenous population in Canada or the United States. The original scoring system of the HFIAS states that affirmative responses to the Worry and Lack Resources items indicate mild food insecurity.[32] The House Empty, Sleep Hungry, and Whole Day Without Eating items are theoretically purported to measure severe food insecurity while the remaining items primarily measure moderate food insecurity. Comparing this scoring system to the difficulty parameters displayed in the current sample in Fig 1, these items appear to function slightly different in the current sample. The Whole Day Without Eating item is associated with a greater difficulty parameter than the other items and thus seems to measure severe food insecurity. Likewise, the difficulty parameter for the Sleep Hungry item indicates that it somewhat taps into the upper level of food insecurity as well. The difficulty parameters for the remaining items appear to cluster together somewhat at an intermediate level of food insecurity, suggesting a degree of overlap and potentially redundancy. It is possible that the cultural differences in the streams of food acquisition affected participants’ response patterns on this scale. Fig 2 displays a model with two items identified as possibly culturally/linguistically inappropriate for this population removed, which increased the average level of information and reduced the overlap between the difficulty parameters for the items. This suggests a potentially stronger model, although removing these items limits our ability to utilize the original scoring system and necessitates the development of a new classification method for this population.

### Strengths

This is the first study in Northern Canada to use a measure that theoretically captures different food sources and unique modes of access in the Inuit food system. Given the culture of Nunavik, and other Arctic regions’ mixed economy, it is particularly important to carefully validate food insecurity instruments locally. Currently, policy responses to food insecurity in Northern Canada are hampered by measurement debates [4], which reinforces the need to validate the tools employed.

This is one of a small number of studies that have investigated food security among pregnant women, a particularly important population given the potential for harm to the developing fetus.[22] Accurate assessment of food insecurity among pregnant women is instrumental in providing culturally appropriate and effective interventions.[29, 30]

This is one of the first studies to utilize a polytomous Rasch model in evaluating a food insecurity questionnaire. By using a polytomous model, we were able to uncover potential problems in their wording and/or format. Other studies that fit food insecurity data to Rasch models first reduced the data to dichotomous variables and thus only were able to distinguish between affirmative versus negative responses.[33] In doing so, they were not able to detect these differences. Furthermore, constructing an empirically-based scoring system using the person parameter estimates from the RSM has likely improved the accuracy with which the HFIAS can measure food insecurity and predict related health problems in this population and others.

### Limitations

Some of the items in the HFIAS were overly long and utilized complex sentence structure. This may have compounded potential concerns about translation issues in Nunavik, where many people speak English as a second language and thus may have had difficulty understanding some questions in the HFIAS.

Because the population of pregnant women in Nunavik during the study period was considerably small, the sample size restricted the types of analyses that could be conducted. Since the sample was relatively demographically homogeneous, we were not able to parse it into groups in a manner that would allow us to perform differential item functioning analyses to accurately establish local independence and measurement invariance. This is recommended for future studies using a more demographically heterogeneous sample. However, these issues were balanced with the need to increase our understanding of the accurate measurement of food insecurity among pregnant women in Nunavik.

Food insecurity measures examine “deficiencies” in food access, availability, quality and/or utilization in a particular population. As all scales can be seen as imperfect measurements of latent variables, we can only assess the extent to which an instrument measures the components of a construct that it directly queries.[8] As such, we cannot assume that the absence of food insecurity, as measured by scales such as the HFIAS, is necessarily food security. This is especially of concern considering that the HFIAS only measures the access component of food insecurity,[26] when there have been concerns raised about the availability, supply, and utilization components of food insecurity in Canadian Inuit populations.[3] While understanding the deficiencies in access to food among such populations is indeed important, such tools are insufficient in understanding the full spectrum of food insecurity/security in these communities.

### Future directions

To establish an accurate food insecurity prevalence rate for this population, it is recommended that future researchers develop a socially and biologically meaningful cutoff. This task should be driven by theory and research on food insecurity in indigenous populations and is beyond the scope of this article. A second recommendation is to develop a scoring program that can facilitate the use of the Theta values among clinicians and public health practitioners.

## Conclusion

The HFIAS shows promise in assessing food insecurity among pregnant women in Nunavik. However, continued modification and validation will help ensure that food insecurity is assessed using the best possible measurement system for this population. Given the predicted prevalence and substantial impact of food insecurity, continued refinement and evaluation of food insecurity measurements is particularly important in this and other vulnerable populations.

## Supporting information

S1 Fig Scree plot for principal component analysis of the HFIAS. The Scree Plot is a graphical representation of the percentage of variance accounted for by each of the first 9 Principal Components. [1] This also shows the large drop between the percentage of variance accounted for from the first to second principal component, suggesting that the items all tap into one latent construct.
(TIFF)

S1 File Food insecurity questionnaire. This is a copy of the food insecurity questionnaire, which includes the HFIAS questions, presented to participants in the AC/DP study.
(DOCX)

S1 Table Principal component analysis of the HFIAS. This table displays the principal components uncovered with the 9 original HFIAS items, representing the underlying components of the data based on an orthogonally-transformed covariance matrix. [1] The eigenvalues display the amount of variance accounted for by each component. [1] The percent of variance accounted for by each principal component is shown in the third column. The final column displays the cumulative percent of variance accounted for by each principal component and the previous components. These results indicate that with six principal components, the model accounts for over 91% of the variance. The first major drop in the amount of variance accounted for by the model occurs between the first and second principal components, suggesting that the scale address a single latent construct.
(DOCX)
