# Supplementary figures and images for: Psychometric validation of the household food insecurity access scale among Inuit pregnant women from Northern Quebec
Source: PLoS One. 2017 Jun 14;12(6):e0178708. doi: 10.1371/journal.pone.0178708 (PMC5470676; doi:10.1371/journal.pone.0178708)

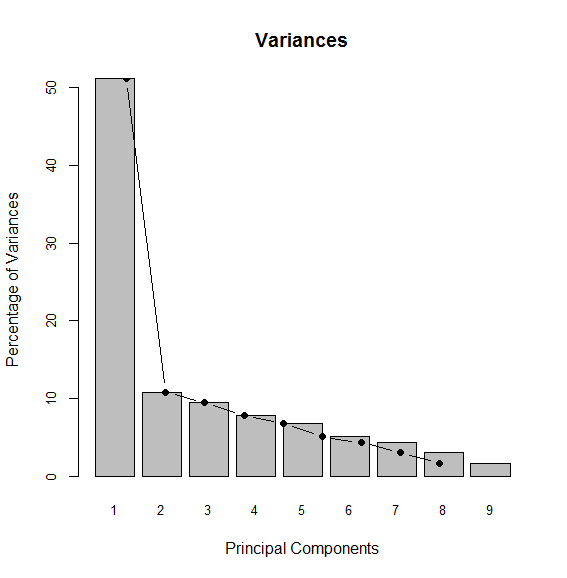

Supplement: S1 Fig — The Scree Plot is a graphical representation of the percentage of variance accounted for by each of the first 9 Principal Components. [1] This also shows the large drop between the percentage of variance accounted for from the first to second principal component, suggesting that the items all tap into one latent construct. (TIFF) [file pone.0178708.s001.tiff]
