# Supplementary material for: Psychometric validation of the household food insecurity access scale among Inuit pregnant women from Northern Quebec
Source: PLoS One. 2017 Jun 14;12(6):e0178708. doi: 10.1371/journal.pone.0178708 (PMC5470676; doi:10.1371/journal.pone.0178708)
Supplement: S1 File — This is a copy of the food insecurity questionnaire, which includes the HFIAS questions, presented to participants in the AC/DP study. (DOCX) [file pone.0178708.s002.docx]

- This section is about *whether you were able to get the food you needed in the past month (30 days) and concerns that you might have had getting the food you needed in the past month.*

- I will read you a series of questions that describe the experiences of some people and families.

- For each question, please tell me whether this happened to you often, sometimes or never in the past month.

(GIVE FOOD SECURITY CARD SO THAT THE RESPONDENT CAN READ EACH QUESTION)

-Questions 1-9 comprise the HFIAS, questions 10-17 are exploratory and were not part of the Rasch analysis

| **In the past month (30 days),…** | | **If yes**: How often did this happen?  *(check the box that applies)* | | |
| --- | --- | --- | --- | --- |
|  |  | **Never** | **Sometimes**  (1-10 days) | **Often**  (>10 days) |
| **1** | Did you worry that you would not have enough food? |  |  |  |
| **2** | Were you not able to eat the kinds of food you preferred because of a lack of resources ? |  |  |  |
|  |  |  |  | |
|  |  |  | | |
| **3** | Did you have to eat a limited variety of foods (e.g. a lot of the same thing) due to a lack of resources? |  |  |  |
| **4** | Did you eat some foods that you really did not want to eat because of a lack of resources to obtain other food items you prefer? |  |  |  |
| **5** | Did you have to eat a smaller meal than you wanted because there was not enough food available for you? |  |  |  |
| **6** | Did you have to eat fewer meals in a day because there was not enough food? |  |  |  |
| **7** | Was your house ever out of food (empty) of any kind because of a lack of resources to get food? |  |  |  |
| **8** | Did you go to sleep at night hungry because there was not enough food? |  |  |  |
| **9** | Did you ever go a whole day and night without anything to eat because there was not enough food in your house? |  |  |  |
| **Please answer the following questions about country foods to the best of your ability.** | | | | |
| **10** | Were you able to get country foods most of the time? |  |  |  |
| **11** | Did you get country foods from a hunter or fisher living in your household? |  |  |  |
| **12** | Did you get country foods from the community freezer? |  |  |  |
| **13** | Did you receive country foods from your friends or relatives not living with you? |  |  |  |

**Please answer the following questions about hunters in your household.**

| **Question** | | **Response** |
| --- | --- | --- |
| **14** | How many hunters do you have in your household? | None End of the questionnaire  1 2 3 4 or more  Answer Q15 to Q17 |
| **15** | In the past month, how many hunters in your household went hunting? | None End of the questionnaire  1 2 3 4 or more  Answer Q16 to Q17 |
| **16** | In the past month, how often did hunters in your household bring home meat? | They were not successful End of the questionnaire  On 1 or 2 days  On 3 or 4 days  On 5 or more days |
| **17** | Did you share in the meat brought home by the hunters? | No  Yes |

Interviewer name : ________________________ Date : ___(dd)/___ (mm)/_______ (yyyy) Time of the day : _______
