# Supplementary material for: Psychometric validation of the household food insecurity access scale among Inuit pregnant women from Northern Quebec
Source: PLoS One. 2017 Jun 14;12(6):e0178708. doi: 10.1371/journal.pone.0178708 (PMC5470676; doi:10.1371/journal.pone.0178708)
Supplement: S1 Table — This table displays the principal components uncovered with the 9 original HFIAS items, representing the underlying components of the data based on an orthogonally-transformed covariance matrix. [1] The eigenvalues display the amount of variance accounted for by each component. [1] The percent of variance accounted for by each principal component is shown in the third column. The final column displays the cumulative percent of variance accounted for by each principal component and the previous components. These results indicate that with six principal components, the model accounts for over 91% of the variance. The first major drop in the amount of variance accounted for by the model occurs between the first and second principal components, suggesting that the scale address a single latent construct. (DOCX) [file pone.0178708.s003.docx]

| Principal Components | Eigenvalues | Percent of Variance | Cumulative Percent of Variance |
| --- | --- | --- | --- |
| 1 | 4.60 | 51.15 | 51.15 |
| 2 | .97 | 10.81 | 61.97 |
| 3 | .85 | 9.44 | 71.41 |
| 4 | .70 | 7.77 | 79.19 |
| 5 | .61 | 6.73 | 85.92 |
| 6 | .46 | 5.09 | 91.01 |

**Reference**

1. Abdi H, Williams LJ. Principal Component Analysis. Wiley Interdisciplinary Reviews: Computational Statistics. 2010;2(4):433-59.
